# From Belly to Brain: Targeting the Ghrelin Receptor in Appetite and Food Intake Regulation

**DOI:** 10.3390/ijms18020273

**Published:** 2017-01-27

**Authors:** Ken Howick, Brendan T. Griffin, John F. Cryan, Harriët Schellekens

**Affiliations:** 1 Department of Anatomy and Neuroscience, University College Cork, Cork, Ireland; k.howick@umail.ucc.ie (K.H.); j.cryan@ucc.ie (J.F.C.); 2 School of Pharmacy, University College Cork, Cork, Ireland; brendan.griffin@ucc.ie; 3 Food for Health Ireland, University College Cork, Cork, Ireland; 4 Alimentary Pharmabiotic Centre (APC) Microbiome Institute, University College Cork, Cork, Ireland

**Keywords:** ghrelin, desacyl-ghrelin, appetite, GHSR-1a, obesity, cachexia, food reward, mesolimbic reward circuitry, blood brain barrier

## Abstract

Ghrelin is the only known peripherally-derived orexigenic hormone, increasing appetite and subsequent food intake. The ghrelinergic system has therefore received considerable attention as a therapeutic target to reduce appetite in obesity as well as to stimulate food intake in conditions of anorexia, malnutrition and cachexia. As the therapeutic potential of targeting this hormone becomes clearer, it is apparent that its pleiotropic actions span both the central nervous system and peripheral organs. Despite a wealth of research, a therapeutic compound specifically targeting the ghrelin system for appetite modulation remains elusive although some promising effects on metabolic function are emerging. This is due to many factors, ranging from the complexity of the ghrelin receptor (Growth Hormone Secretagogue Receptor, GHSR-1a) internalisation and heterodimerization, to biased ligand interactions and compensatory neuroendocrine outputs. Not least is the ubiquitous expression of the GHSR-1a, which makes it impossible to modulate centrally-mediated appetite regulation without encroaching on the various peripheral functions attributable to ghrelin. It is becoming clear that ghrelin’s central signalling is critical for its effects on appetite, body weight regulation and incentive salience of food. Improving the ability of ghrelin ligands to penetrate the blood brain barrier would enhance central delivery to GHSR-1a expressing brain regions, particularly within the mesolimbic reward circuitry.

## 1. Introduction

Food intake is one of the most deceptively complex of all mammalian behaviours, being regulated by a variety of homeostatic and external factors [1]. One of the key hormones regulating food intake is ghrelin, a 28 amino acid (aa) peptide synthesized and secreted by gastric oxyntic cells [2]. Blood levels of this hormone exhibit circadian fluctuation which are aligned with mealtimes, spiking pre-prandially followed by rapid post-prandial reductions [3]. These have positioned ghrelin as a key regulator of meal initiation, stimulating a cascade of events to prepare the body for an impending meal. All of the above has led to the designation of ghrelin as the “hunger hormone” [3], although recent findings provide evidence for compensatory mechanisms in ghrelin knockouts [4]. To date however, it remains the only known peripheral hormone with orexigenic effects via a centrally mediated mechanism [5,6,7]. The genes and cellular mechanisms involved in the synthesis, cleavage and octanoylation of ghrelin have been extensively described [8,9,10,11]. Briefly, the addition of an acyl functional group to the serine-3 of ghrelin is essential for its binding to and activity on its receptor, the growth hormone secretagogue receptor (GHSR-1a) [2]. The neuronal pathways underlying ghrelin’s appetite-stimulating effects centre around activation of the GHSR-1a, which is abundantly expressed in the hypothalamus [12,13]. The arcuate nucleus is the main hypothalamic locus for ghrelin’s orexigenic effect. GHSR-1a-mediated activation of arcuate nucleus neuropeptide Y/agouti-related peptide neurons serves to stimulate orexigenic activity through Y1 receptors, while concomitantly inhibiting satiating pro-opiomelanocortin neurons [14]. Furthermore, ghrelin contributes to the regulation of body weight by potently stimulating growth hormone (GH) secretion from the pituitary, increasing adiposity and reducing energy expenditure [10,15]. Goldstein and Brown showed that ghrelin-stimulated GH secretion is critical to protecting the body from starvation-induced hypoglycaemia [16]. Ghrelin has also been shown to be involved in reward processes, mood, memory and learning, and stress response [17,18,19], while peripheral functions span gastric motility, glucose homeostasis, immune function, cardiac output and bone formation [20,21,22,23,24,25].

### 1.1. Ghrelin and Appetite-Related Disorders

In appetite and food intake, ghrelin’s role can be subdivided into two distinct, yet overlapping areas of homeostatic and non-homeostatic feeding [26,27,28,29,30]. The term “non-homeostatic” encompasses both motivation and incentive salience applied to food rewards, but also the inherent palatability or “hedonic” aspect of eating in itself. The ghrelin system not only acts as a barometer for energy balance [5,10], but also contributes to the drive for eating beyond metabolic demand and the consumption of palatable foods [28,29]. Therefore, ghrelin and the GHSR-1a receptor, have been extensively investigated as potential therapeutic targets to tackle metabolic, eating- and appetite-related disorders by virtue of the unique position which the ghrelinergic system occupies at the interface of homeostatic and hedonic feeding.

### 1.2. Homeostatic Feeding

The ghrelinergic system has received considerable focus as a target in maladaptive changes to homeostatic energy balance [10,31,32]. This is achieved through manipulating a number of physiological mechanisms resulting in a net anabolic effect in the body [14,33]. The normal ageing process yields a number of physiological changes which lead to a reduction in appetite and appropriate nutritional intake [34,35]. Declining ghrelin levels contribute to this reduction in food intake and lean body mass [34]. Furthermore, ageing population demographics translate to a greater incidence of chronic conditions such as cardiovascular disease, respiratory disease and cancer [36]. Chronic diseases compound a weakening ghrelin axis by increasing systemic inflammation and cytokine output [37]. Cytokine-mediated activation of anorexigenic neuron populations in the hypothalamus causes a cascade of metabolic changes resulting in loss of lean and fat mass, and the development of cachexia [34,35,38]. Thus, a metabolic backdrop is created which antagonises ghrelin’s somatotrophic effect [37,38,39]. Age-related malnutrition and under-eating following chronic diseases results in prolonged hospital stays, decreased independence and poorer response to treatment, leading to a greater burden on global health infrastructures and poorer clinical outcomes [34,35,40].

### 1.3. Non-Homeostatic Feeding

Further to its role as a key mediator of the energy balance “set point”, ghrelin is also implicated in incentive salience and motivation to eat, and consequently has become a therapeutic target for development of therapies for overeating and obesity [41,42]. The need for anti-obesity therapeutics is highlighted by the global increase in incidence of obesity in recent years. In 2014, more than 1.9 billion adults (39% globally) were overweight [43] and obesity continues to rise to epidemic proportions. In Western society particularly, consumption of readily available high-fat and high-sugar meals, together with increasingly sedentary lifestyles, has led to a rise in the “metabolic syndrome”. This is a condition associated with weight gain, hyperglycaemia, insulin resistance, hypercholesterolaemia and a general inflammatory phenotype [44,45]. In addition to homeostasis, neuronal pathways also exist which promote the consumption of palatable, calorie-dense foods beyond the metabolic demands of the organism [46]. This is thought to be an evolutional mechanism that promotes over-eating of calorie-dense foods in preparation for times of food deprivation. Needless to say, this is redundant in the western world where there is an abundance of food. The mesolimbic dopaminergic pathway in the brain is known to be a key mediator in this primitive drive [47,48,49]. Overconsumption of palatable foods is thought to be triggered by hyperactivity of the reward system [50,51]. Furthermore, the late Bart Hoebel and colleagues in Princeton proved that sugar in itself can share many of the properties of addictive substances [52,53]. In fact, palatable foods are now known to share the same reward pathways as non-psychostimulant drugs of abuse [54]. It should be noted that although the concept of food addiction has gained significant ground, it has many heuristic limitations [55,56].

Increases in circulating levels of endogenous ghrelin, following periods of food restriction, signal an increase in appetite and hunger and are correlated with a general increase in both “liking” and “wanting” of food [57,58]. Interestingly, the elevated endogenous ghrelin levels have been associated with an increased dopamine output in the brain [59], while functional magnetic resonance imaging in human subjects has shown that ghrelin administration enhances the activation of the central reward circuitry in response to images of pleasurable foods [60,61]. Subsequently, ghrelin’s role in increasing the incentive valuation of food at the level of the mesolimbic circuitry has come to the fore in recent reviews [57,62].

### 1.4. Stress, Impulsivity and Cognition

Dopaminergic activity in the mesolimbic reward circuitry not only increases perceived rewarding value of food, but also results in increased impulsive action [63]. Impulsivity can be defined by characteristic motor disinhibition and impaired decision-making, and a strong correlation exists between impulsiveness and food reward behaviour [64,65,66]. Furthermore, a relationship between increased ghrelin levels and impulsive behaviour has recently been elucidated [67]. Therefore, dysregulation of ghrelin-dopamine signalling is thought to contribute to the development of an addictive-like relationship with food. Additionally, numerous groups have published on the ghrelin system linking stress, mood and food reward. Food intake and choice of food are closely linked with how we deal with stress [68,69,70]. This is becoming increasingly pertinent in modern society due to the combination of readily available high-calorie foods, and the exposure to chronic stressors [71,72]. Ghrelin is known to play a role in stress-induced food intake and the phenomenon of “comfort eating” [18,73,74]. A combination of low impulse control and increased incentive to eat palatable foods synergistically contribute to the development of obesity [75].

### 1.5. Current Status and Implications

Consequences of over- and under-eating constitute ever-expanding health problems that remain unanswered in modern society, despite education, public health campaigns and pharmacotherapy [76,77]. Thus, there is an impetus to understand the physiological mechanisms underlying central appetite regulation and food intake in order to design novel treatment strategies for eating disorders. However, despite almost 20 years since it is discovery by Kojima and colleagues, no specific ghrelin targeting anti-obesity drug or cachexia therapeutics are on the market for clinical use [2]. The literature on ghrelin illustrates a plethora of information, yet we are still faced with a paucity of success. As knowledge on ghrelin increased, the role of the hormone shifted from the key protagonist in feeding initiation to be considered as part of a spectrum of diverse physiological processes. The peripheral and central distribution of the GHSR-1a and the heterogenous nature of GHSR-1a signalling result in pleiotropic actions of ghrelin, many of which are still being investigated.

In this review we discuss the distribution and heterogenous signalling of the GHSR-1a, and its relevance to ghrelin’s action. Furthermore, we review the pharmacokinetics and pharmacodynamics of both native ghrelin and synthetic ghrelin ligands used clinically to date, and propose that augmenting their blood brain barrier (BBB) penetrability would better target the GHSR-1a at the level of feeding and reward centres in the brain, thus increasing specificity for appetite-modulation and limiting off-target peripheral tissue effects.

## 2. Growth Hormone Secretagogue Receptor (GHSR-1a) Receptor—Biodistribution and Signalling

### 2.1. Pleiotropic Pharmacodynamics

**Figure 1:**
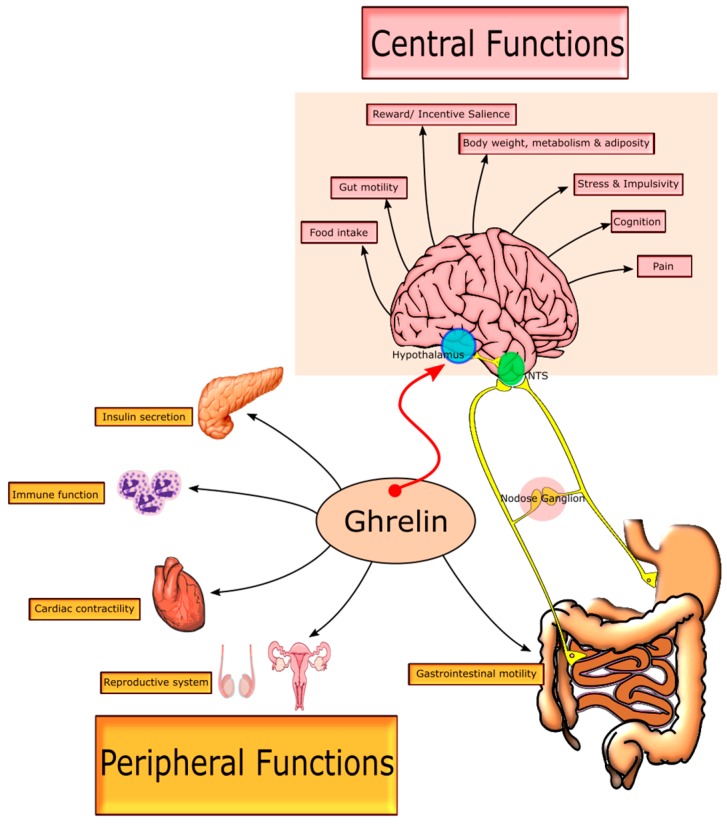
This combines the documented methods of ghrelin’s action after its release from the stomach, or exogenous administration. Ghrelin travels via the circulation to activate the growth hormone secretagogue receptor (GHSR-1a) in the arcuate nucleus and the nucleus tractus solitarius (NTS) after circumventing the blood-brain barrier (BBB), denoted by the red arrow. Peripheral signals are conveyed to the central nervous system (CNS) via vagal afferents also. Activation of the GHSR-1a leads to a multitude of centrally and/or peripherally mediated effects.

The target for ghrelin and ghrelin ligands is the GHSR-1a receptor, a 7 transmembrane G-protein coupled receptor (GPCR). The GHSR-1a receptor is expressed both in the central nervous system (CNS) and peripherally in the body, and binding of acyl-ghrelin leads to receptor activation [2]. The distribution of the GHSR-1a receptor is of paramount importance as it is the executor of ghrelin’s function. Indeed, it is the peripheral (exclusive to non-CNS tissue) and central (exclusive to the CNS) distribution of the GHSR-1a which is responsible for the plethora of physiological effects which ghrelin exerts (Figure 1). The GHSR-1a is densely expressed in the hypothalamic nuclei which sends neuronal projections to other appetite regulating centres [13,78]. Peripherally, GHSR-1a is located on vagal afferents, pancreatic cells, spleen, cardiac muscle, bone, adipose, thyroid, adrenal glands and on immune cells [13,79]. Therefore, given the ubiquitous expression of the receptor, any instance of exogenous ghrelin or ghrelin ligand administration leads to a combination of downstream effects. Neither exogenous ghrelin nor ghrelinergic compounds can effectively target centrally-controlled food intake, without affecting a multitude of other central and peripheral outputs [7,42]. The non-specific tissue effects of peripheral ghrelin administration may be further complicating an intricate metabolic balance and need to be considered.

### 2.2. Central GHSR-1a Signalling

Food intake, adiposity and energy homeostasis are centrally controlled functions of ghrelin and the GHSR-1a which have been extensively described in the literature [5,10,14]. Chronic central administration of ghrelin induces adiposity in rodents by reducing the utilization of fat as an energy substrate [10]. Further work confirmed this central action, with expression of mRNA for fat-sparing enzymes fatty-acid synthase, acetyl-CoA carboxylase α, stearoyl-CoA desaturase-1, and lipoprotein lipase all being increased with chronic intracerebroventricular infusion of ghrelin. In addition, mRNA expression for carnitine palmitoyltransferase-1α, involved in fat utilisation is decreased while lipid mobilization is reduced following ghrelin treatment, as shown by an increase in respiratory exchange ratio in vivo [80,81]. Furthermore, ghrelin stimulates lipid deposition in human visceral adipose tissue in a dose-dependent manner [82]. Acute ghrelin administration consistently stimulates food intake across species [3,41,58,83,84,85,86,87,88]. In recent years however, research has proven that ghrelin may not be the critical regulator of food intake it was once heralded to be.

Studies in knockout mice have confirmed the ghrelin peptide is not a key mediator of food intake or growth [89]. In contrast with predictions, ghrelin knockout mice are neither undersized nor hypophagic; their behavioural phenotype for food intake and physical attributes are indistinguishable from wild-type littermates [4,89]. Ghrelin-null rodents also display normal responses to starvation and diet-induced obesity [89]. Furthermore, ablation of ghrelin in adulthood failed to elicit effects on food intake, body weight, or resistance to diet-induced obesity [4]. Interestingly, both germline ghrelin-deficient and ghrelin cell-ablated mice display a profound hypoglycaemia following prolonged calorie restriction. Overall however, the phenotype in ghrelin-knockouts is suggestive of a non-critical role for ghrelin in food intake and growth.

Despite the apparent compensatory mechanisms that exist in the absence of ghrelin, exogenous ghrelin or ghrelin ligands have the potential to significantly modulate appetite, most likely via central GHSR-1a signalling. Recently it was shown through neuronal-specific ablation of the GHSR-1a that receptor signalling within the CNS is a crucial regulator of energy metabolism. This is important to consider in the context of the high constitutive activity of the GHSR-1a, which does not require ghrelin in order to become activated [90,91]. Zigman and colleagues, amongst others, have demonstrated that GHSR-1a-null mice are resistant to diet-induced obesity [92,93,94]. Neuronal GHSR-1a is also essential for ghrelin-induced meal initiation and maintenance of body weight in conditions of caloric deficit [95]. Central GHSR-1a signalling therefore seems to be critical for not only acute initiation of food intake, but also is a key mediator of body weight. Supporting this, a genetic mutation in GHSR-1a that allows ghrelin binding but prevents activation of the receptor, leads to the condition of familial short stature [96].

Consistent with the notion of a multifunctional role for ghrelin, the GHS-R1a receptor is also expressed in several non-hypothalamic brain areas. In-situ binding studies have demonstrated the existence of the GHSR-1a in the midbrain dopamine system, particularly the main mesolimbic reward circuitry structures; the ventral tegmental area (VTA) and its primary projection site, the nucleus accumbens [12,17,97]. The VTA projects GHSR-1a-expressing dopaminergic neurons which terminate in the nucleus accumbens (NAcc), a hotspot for dopamine release which is critically associated with promoting incentive value of drugs of abuse and natural rewards, including food [98]. Further projections from the VTA to the medial prefrontal cortex, an important part of the reward system which also encodes the genes for the GHSR-1a, are described as part of this pathway [99,100,101]. Consequently, the GHSR-1a located in the midbrain dopaminergic pathway may be a driver for the decision to eat palatable, calorie-dense foods, irrespective of metabolic need.

GHSR-1a receptor is also expressed in areas associated with memory, emotional arousal and cue-potentiated feeding [7,102,103]. For example, GHSR-1a in the hippocampus is known to play a role in synaptic plasticity, increasing hippocampal spine density and enhancing long-term potentiation, an important phenomenon in learning and memory consolidation [102]. Activation of hippocampal GHSR-1a in vivo increased performance and retention of memory-dependent tasks [19,102]. Furthermore, the GHSR-1a is densely expressed in several sub-nuclei of the amygdala and is associated with amelioration of anxiety-like behaviours in food scarcity [104]. Altogether, the above is supportive of a broader, non-homeostatic function for GHSR-1a signalling in higher brain functions dependent on metabolic status, for example, heightened salience and increased memory consolidation in times of hunger to remember where food can be obtained [102]. Critically, although ghrelin peptide mRNA is not found in the brain, it’s expression is noted peripherally, suggesting multiple potential autocrine or paracrine roles of the hormone [13,105,106]. Indeed, direct actions of ghrelin in the periphery have been reported in several organ systems.

### 2.3. Peripheral GHSR-1a Signalling

The GHSR-1a is responsible for several peripheral mechanisms modulated by ghrelin including, but not limited to, cardiac contractility, bone formation and reproductive function. Firstly, GHSR-1a is expressed on rodent and human immune cells, including monocytes and T cells [13,20]. Ghrelin and ghrelin agonists have shown a protective effect under acute endotoxaemia, enhancing the effectiveness of immune response through tissue infiltration in vivo [22,23], leading to decreased mortality. Ghrelin is also known to directly reduce the expression of inflammatory cytokines [20]. Secondly, protective effects have also been attributed to ghrelin in rodent cardiomyocytes [24,107]. The cardioprotective mechanisms underlying this have been described in detail elsewhere [108]. The ghrelin agonist, hexarelin, was shown to increase cardiac output in rodents and humans [85,109]. Thirdly, ghrelin and the GHSR-1a receptor are expressed in rat and human testis [13,110,111] and in females both have been documented to be expressed in ovary, hilus cells (leydig cells) and corpora lutea, all of which are hormone secreting cells which play roles in the female reproductive cycle [25]. Ghrelin plays a crucial role in the regulation of the hypothalamic-pituitary-gonadal axis mainly through reducing secretion of hypothalamic gonadotropin-releasing hormone and stimulating local luteinizing hormone and follicle stimulating hormone secretion.

### 2.4. Complementary Signalling: Gastrointestinal Motility, Glucose Homeostasis and Visceral Pain

All of the above have discussed distinct centrally-mediated and non-central autocrine or paracrine functions of GHSR-1a. In certain instances, central and peripheral ghrelinergic signalling appear to be complementary, as is the case for regulation of gastrointestinal motility, glucose homeostasis and visceral pain. The role of ghrelin and the GHSR-1a in the regulation of gastrointestinal tract motility has already been reviewed [112]. The GHSR-1a receptor is located in the mucosa and myenteric plexus of rodent and human gastrointestinal tract, reinforcing the local neural role for ghrelin in gut motility [113,114,115]. In vitro, this notion was supported by contractility studies showing that ghrelin directly activates both cholinergic [114,116,117] and tachykinergic excitatory neurons in fundus and antrum. In vivo, peripheral administration of ghrelin accelerates gastric emptying in a dose-dependent manner [117,118,119,120]. In humans, ghrelin infusion stimulates gastric emptying in healthy participants and ameliorates symptoms of gastroparesis [121]. However, central administration also displays a pronounced effect on gastrointestinal tract motility [122,123]. Vagotomy or chemical deactivation of the vagus were shown to abolish the observed effects of peripherally administered ghrelin [116,124]. Ghrelin’s effects in respect of gastrointestinal motility thus seem to be vago-vagal in origin—meaning that it results from reciprocal vagal communication between the gut and the dorsal vagal complex of the brain. Similar to food intake and adiposity above, gastric emptying is unaffected in ghrelin knockout rodents, suggesting the existence of compensatory mechanisms [112]. Critically, it has been suggested that local mechanisms become operational under abnormal conditions such as vagal denervation or pharmacological stimulation [122]. Supporting this, it was shown that downregulation of GHSR-1a in the small intestine delays transit in vagotomised mice [125]. Overall, evidence suggests that ghrelin acts from the periphery in a remote fashion to modulate gastrointestinal function from the CNS via the vagus nerve, however the gastrointestinal distribution of the GHSR-1a paves the way for local activity which may be heightened by pharmacological stimulation [122]. The motilin receptor has also been characterized in the human gastrointestinal tract [126] and displays close structural homology and a functional compensatory role with the GHSR-1a in gastrointestinal motility [127].

Interacting central and peripheral GHSR-1a signalling is evident in the physiology of glucose homeostasis. Many peripheral hormones act in a central manner to regulate energy metabolism and glucose balance, including glucagon, glucagon-like peptide 1 and insulin [128,129,130,131]. However, the GHSR-1a is expressed in pancreatic α and β cells [113,132,133,134], and peripheral ghrelin acts directly on the receptor in pancreatic islets to modulate the release of insulin [132,135,136]. In humans, Broglio and colleagues found that acute administration of acyl-ghrelin in the fasted state significantly reduced plasma insulin while promoting hyperglycaemia, however, a continuous infusion stimulated insulin secretion secondary to elevated glucose levels [137,138]. Supporting this, several studies have consistently shown that ghrelin administration promotes hyperglycaemia [139]. Central administration of ghrelin also regulates plasma insulin in rodents [140,141,142,143]. Somewhat confusingly, it seems that central GHSR-1a signalling exerts an insulinotropic effect, versus the inhibition of glucose-stimulated insulin secretion by peripheral GHSR-1a activation [135,142], meaning that the receptor may play distinct roles in glucose homeostasis depending on the site of action. Furthermore, administration of acyl-ghrelin into the portal, but not the femoral vein inhibited glucose-stimulated insulin secretion. Hepatic vagotomy attenuated this inhibition suggesting indirect central control over insulin secretion via neural signalling [144,145]. Critically, fasting decreases insulin levels in both wild type and ghrelin knockouts, as well as producing comparable responses to both hypo-caloric and hyper-caloric situations. Hence, compensatory pathways seem to exist for glucose homeostasis, however GHSR-1a knockout leads to reduced glucose levels under calorie- deprivation [89,146]. Later work from the same group used GHSR-1a-null mice to show reduced adiposity and insulin resistance [147]. A body of evidence thus exists to support the indirect central control of GHSR-1a signalling over glucose homeostasis. Furthermore, it seems that metabolic status is a key determinant of the regulatory action of central ghrelin on peripheral glucose homeostasis [143]. A recent review summarized the complex interrelationship that exists between ghrelin, insulin and glucose [148]. The ability of insulin and glucose levels to appreciably impact on appetite [149] means that indiscriminate targeting of the GHSR-1a without due consideration of the effects on peripheral glucose and insulin metabolism may ultimately decrease efficacy of appetite modulation therapy [150,151].

Ghrelin and the GHSR-1a has also been the subject of investigation in the modulation of pain transmission [152]. Originally, ghrelin’s role in pain sensitivity was thought to be through a combination of central and peripheral GHSR-1a signalling [153,154]. Chronic peripheral ghrelin administration has been shown to attenuate neuropathic pain in rats [155]. Ghrelin treatment resulted in elevated levels of anti-inflammatory cytokines in vivo in a rodent model of inflammatory pain [156]. It has also been shown that central and peripheral ghrelin administration prevents the pain response response caused by intraplantar insult [157]. Furthermore, mRNA for GHSR-1a is found in pain-processing centres including the sensory motor cortex and the dorsal horn of the spinal cord [154,158,159,160]. Current opinion seems to agree that ghrelin’s analgesic effect is conveyed mainly through central mechanisms, via interactions with the opioid system [152,157,161,162]. Therefore, ghrelin and the GHSR-1a may have communicating peripheral and central pathways in the modulation of pain sensitivity.

### 2.5. Heterogenous Action—GHSR-1a as a Promiscuous Target

Further to the distribution of GHSR-1a and the consideration of central and peripheral effects, the receptor is known to display heterogenous signalling cascades, downregulation/internalization and heterodimerization—all of which are akin to other GPCR’s and constitute important considerations for appetite modulation therapy [163]. Downstream effects of the GHSR-1a via coupling to different G-proteins have been reviewed in detail elsewhere [26]. Importantly, it is worth emphasising that the GHSR-1a displays heterogenous functions dependant on the location of the receptor expression in the body. For example, in neurons of the arcuate nucleus, ghrelin acting on the GHSR-1a induces orexigenic neuropeptide Y release through N-type voltage-gated Ca_2β_ channels via cyclic adenosine monophosphate (cAMP) increases in the cell [164]. In pituitary cells responsible for effecting somatotrophin release, GHSR-1a mainly acts via G_αq_ coupled G-protein to trigger calcium release from intracellular stores [165]. These signalling pathways are both excitatory—interestingly, in the periphery, ghrelin binding to GHSR-1a in pancreatic β cells leads to an inhibition of cAMP and hyperpolarization of the cell [166].

The GHSR-1a not only exhibits site- and ligand-dependant signalling; it demonstrates an ability to “cross-talk” with other neuroendocrine GPCRs [167]. The receptor has been shown to pair or dimerize with other receptors, leading to either attenuation or augmentation of signalling. GHSR-1a: melanocortin-3 receptor protomers have been described; melanocortin-3 receptor is an important downstream signalling receptor in the homeostatic control of food intake [168]. Rediger and colleagues showed that the signalling modalities of one GPCR was dependent on the conformational activity of the other. In essence, ghrelin-induced GHSR-1a activation is attenuated by interaction with the melanocortin-3 receptor [169]. We previously demonstrated the existence of GHSR-1a: Serotonin 2C dimers in vitro, hypothesizing novel pharmacological targets for drug treatment based on the involvement of serotonin 2C receptor in satiety signalling [167,170,171,172]. Furthermore, GHSR-1a: Dopamine D2 receptor co-expressed on neurons leads to attenuated dopaminergic response upon administration of a GHSR-1a antagonist in vivo [173]. Critically, it is the allosteric interaction of the GPCR protomer which results in the observed cross-talk, rather than the net effect of independent neuroendocrine signalling [173]. More recently, it was shown that hippocampal-dependent synaptic plasticity is modulated by GHSR-1a: Dopamine D1 heterodimerization [103]. Moreover, an inactiveisoform of GHSR-1a receptor, the GHS-R1b, is worthy of mention here though it is not a major focus of review. GHSR-1b is a truncated, 5-transmembrane receptor [174]. The GHSR-1b receptor exhibits widespread tissue distribution and exhibits an ability to co-localize with the GHSR-1a receptor causing a subsequent attenuation of activity through an increased internalization of the active receptor. This is potentially significant in the backdrop of ghrelin signalling as the GHSR-1a exhibits high constitutive signalling in the absence of its native ligand [90,91,175].

As well as heterogenous signalling and neuroendocrine cross-talk, the expression of the GHSR-1a on the cell membrane is critical to it being a successful therapeutic target. However, GPCRs are known to downregulate via receptor internalization or endocytosis causing a subsequent attenuation of effect [176]. Unsurprisingly, the GHSR-1a receptor has been shown to downregulate in response to various stimuli, including ghrelin- and ghrelin-ligand mediated activation [177,178,179]. After binding of ghrelin to GHSR-1a, the complex is internalised in clathrin-coated pits, from which the receptor needs to be recycled back to the surface of the cell [178]. In vitro growth hormone release is rapidly desensitized after exposure to a ghrelin agonist, MK-0677, and in vivo response in beagles was reduced to 25% after 4 days of daily administration [180]. In line with this, growth hormone release declines rapidly upon repeated ghrelin administration in humans [181]. There is a dearth of information in the literature to suggest an ability of ghrelin to sustain elevated food intake in animals or humans upon long-term administration, and it is feasible that downregulation would contribute to a decline in orexigenic effects over time. One study showed no overall effect on food intake in rats after chronic administration of acyl-ghrelin [81]. A limited number of clinical studies have failed to show an appreciable difference in food intake with chronic administration of ghrelin [182] or the synthetic agonist growth hormone releasing peptide-2 [86]. However, in acute situations consistently pronounced orexigenic effects are reported in both animals and humans [58,87,183,184]. Conversely, GHSR-1a has been shown to upregulate, in the hypothalamus at least, during fasting [90]. Hence, GHSR-1a expression levels, and subsequent effect of receptor modulation, are heavily dependent on the metabolic state. To further confirm this, it has been noted that leptin-deficient Zucker rats, characterized by profound hyperphagia, display a heightened expression of the GHSR-1a and a corresponding increased sensitivity to ghrelin and ghrelin agonists [185].

In summary, the above described heterogeneity of the GHSR-1a in terms of distribution, downstream signalling, tachyphylaxis and neuroendocrine communication paints a complex picture. This complexity has hindered development of an effective GHSR-1a targeting therapy for appetite modulation. It seems that the effect of GHSR-1a modulation hinges on the metabolic backdrop in which the therapy is delivered, hence the indiscriminate targeting of the GHSR-1a with non-specific systemic delivery of varying ligands may be one of the reasons for a lack of efficacy to date. The widespread nature of the receptor in the body leads to GHSR-1a activation in off-target sites, potentially leading to local effects which can ultimately inhibit the intended benefit.

## 3. Ghrelin and Ghrelin Ligands: Pharmacokinetic Perspectives

On the whole, central action seems to be critical for GHSR-1a-mediated appetite modulation and energy balance. Understanding the pathway by which peripheral ghrelin acts centrally, after either endogenous release or exogenous administration, is critical to achieving therapeutic exploitation. As mentioned earlier, the question of whether ghrelin peptide is expressed in the brain is controversial and the subject of debate. Ghrelin immuno-reactive cells have been reported in the hypothalamus in some studies [14,186], while the existence of ghrelin-producing cells was reported in the arcuate nucleus of the hypothalamus [187]. Recent evidence seems to refute these claims and now it is thought ghrelin is only present in these areas due to access of circulating ghrelin from the periphery [57,106,188]. The main pathways by which ghrelin is thought to exert its orexigenic effect after it is released from the stomach have been extensively reviewed [42].

### 3.1. Blood Brain Barrier Penetration

The orexigenic effects of ghrelin have immediate onset, with food intake increasing 10 min after systemic administration [3,188]. It follows therefore that ghrelin must have ready access into the brain. In fact, ghrelin can directly cross the blood brain barrier (BBB) at areas which are not highly protected, and subsequently convey its effect via neural projections from the site of entry to various feeding centres [78,189]. This is supported by the suggested “leaky” nature of the BBB surrounding the circumventricular organs of the brain [190,191,192]. The fenestrated endothelia surrounding the hypothalamus are supplied by capillaries which confer a rich blood supply, allowing the hypothalamus to sample the contents of the systemic circulation [193]. This affords many central nervous system (CNS) active peptides, including ghrelin, access to the CNS while still retaining effective and selective barrier function for the brain [12,194]. Furthermore, the blood—cerebrospinal fluid (CSF) barrier which exists at the choroid plexus also has been shown to allow ghrelin access to the arcuate nucleus. This is composed of a differentiated layer of cells that surround a core of capillaries in some brain ventricles and produce CSF, and/or the hypothalamic tanycytes, a specialized layer of bipolar ependymal cells that line the floor of the third ventricle and bridge the CSF and the capillaries of the median eminence [195,196]. Other circumventricular organs such as the area postrema, a part of the dorsal vagal complex, affords ghrelin diffusive access to the abundance of GHSR-1a’s in the nucleus tractus solitarius and dorsovagal nucleus. The nucleus tractus solitarius (NTS) is a relay hub for appetite regulation with a complex network of efferent and afferent connections. The NTS converts humoral responses into neuronal communication [197].

### 3.2. Vagus Nerve Signalling

The NTS is also important to the other described route by which peripheral ghrelin accesses central GHSR-1a; remote modulation from the gut signalling through the vagus nerve and the brainstem [42,113]. Indeed, several gastrointestinal hormones such as cholecystokinin (CCK), peptide YY, and glucagon-like peptide 1 (GLP-1), transmit orexigenic and satiating signals to the brain, at least in part, via vagal afferents [198,199,200]. Feeding-related information can travel directly to the dorsal vagal complex and NTS, where signals are converted from humoral to neural format and further relayed to higher brain levels. Indeed, it is known that gut derived peptides such as the satiating CCK exert their central action via vagal afferents from the gastrointestinal tract [201]. Early studies using c-Fos expression as a marker of neuronal activation showed that peripheral administration of a ghrelin mimetic increased Fos protein in the NTS [202]. The NTS provides a direct noradrenergic projection to the hypothalamus which is believed to be important for neural regulation of energy balance and food intake [203]. Date and colleagues demonstrate that peripheral ghrelin signalling reaches the NTS by either blood or neural mechanisms and relays noradrenergic stimuli to the hypothalamus to increase feeding [113,204,205]. Transections above the level of the NTS, or specific ablation of dopamine β-hydroxylase (the noradrenaline synthesizing enzyme), abolished peripheral ghrelin-induced feeding [205]. Moreover, it has been reported that the orexigenic action of ghrelin is attenuated in humans who underwent gastric surgery involving complete or partial vagotomies [206]. Vagotomy also abolishes the orexigenic activity of ghrelin in rats [113]. Another preclinical study however, reports that ghrelin’s orexigenic effect remains intact after a subdiaphragmatic vagal deafferentiation. The authors argue that a bilateral vagotomy, as described in Date’s work, would indiscriminately remove both afferent and efferent vagal innervation, thereby severing a multitude of other physiological processes, including satiating signals [207]. It is thus stated that subdiaphragmatic vagal deafferentiation is a more representative model for ablating the vagal afferent connection as it is less invasive to other vagally-mediated physiological parameters such as heart rate and respiration. However, the dose of ghrelin used in this study was substantially higher than that used in the original work by Date therefore results cannot be directly compared. Critically, it points to the fact that vagal signalling is not essential to relay ascending orexigenic messages, likely due to the fact that the area postrema can facilitate diffusive access of ghrelin from the bloodstream to the NTS, enabling ascending signalling even without vagal innervation of the NTS. This is supported by the fact that intravenous ghrelin administration stimulates growth hormone secretion in vagotomised patients [208]. Taken together, all of the above information strongly suggests an interlinked role between blood and neural pathways for conveying ghrelin’s signal from the periphery to the CNS.

### 3.3. Ghrelin Human Studies

Normal serum ghrelin levels vary in man and reach 0.2–0.4 pmol/mL in hunger states [58,209], with active ghrelin levels peaking at of 0.01–0.035 pmol/mL [210,211,212,213]. Intravenous infusions of 1–40 pmol/kg/min active ghrelin have been used clinically to increase appetite acutely in cachectic states [58,121,184,214,215]. From a pharmacological perspective doses in this range are supraphysiological and have resulted in several hundred-fold changes in both active and total plasma ghrelin (Table 1). Lippl and colleagues administered doses of ghrelin more representative of the levels experienced endogenously, resulting in active ghrelin increasing to 0.057 pmol/mL (2.4-fold increase from baseline) [216]. This elevation failed to show an orexigenic effect in participants [216]. Critically, endogenous active ghrelin reaches similar levels after overnight fasting (0.1–0.35 pmol/mL) [58,209,217], predictably stimulating food intake and increasing incentive salience of food [14,58]. However, higher levels of plasma active ghrelin (>1.6 pmol/mL) have been required to produce an appetite-stimulating effect in clinical studies [58]. This may be indicative of the fact that many studies administer ghrelin in fasted states, therefore necessitating a higher dose in order to overcome elevated basal ghrelin levels. Indeed, Lippl and colleagues was the only study which administered ghrelin in the fed state to patients, and therefore had low basal levels of ghrelin (Table 1). It also may be a reflection that many studies fail to account for desacyl-ghrelin. This was originally thought to be a pharmacologically inactive breakdown product of active ghrelin but recent evidence has shown this is not the case [218].

### 3.4. Acyl and Desacyl-Ghrelin—Implications for Therapeutic Approaches

Both acylated and desacyl-ated forms of the hormone ghrelin are detected in the peripheral circulation [224]. Despite this, many studies assessing ghrelin levels in blood fail to specify the acylation status of the hormone [225]. In fact, only some preclinical studies have distinguished between the effects of acyl- and desacyl-ghrelin [226,227,228]. Furthermore, it is critical for accurate measurement of acyl- ghrelin that blood samples are appropriately stabilized in order to prevent desacyl-ation [218,229]. The binding of acyl-ghrelin and subsequent activation of GHSR-1a is well established [2,230]. Similarly, the lack of desacyl-ghrelin binding to GHSR-1a is described [2]. Desacyl-ghrelin does not compete with acyl-ghrelin for GHSR-1a binding at physiological concentrations [231], however, it has been shown to activate the receptor at supraphysiological concentrations [142,232]. Desacyl-ghrelin is the most abundant form in the circulation and is purported to be the active ligand for additional, as yet unknown, GHSR subtypes [26,218,233].

Peripheral acyl-ghrelin administration markedly increases circulating GH, prolactin, adrenocorticotrophic hormone, and cortisol levels [233]. This is accompanied by a decrease in insulin and a concomitant increase in plasma glucose. Interestingly, although desacyl-ghrelin administration had no such effects in isolation, when administered in combination with acyl-ghrelin it was able to negate the observed effects on plasma insulin and glucose [233]. Indeed, it has been suggested that desacyl-ghrelin should be considered as a hormone distinct from acyl-ghrelin given its ability to elicit effects on certain peripheral actions such as cardiovasculature, cell proliferation and certain aspects of adiposity [233]. Overnight intravenous desacyl-ghrelin infusion was found to improve glucose metabolism and, conversely to acyl-ghrelin, display a glucose-lowering effect [234]. Moreover, combined administration of acyl- and desacyl-ghrelin strongly improved insulin sensitivity compared to acyl-ghrelin administration alone [235]. Therefore, desacyl-ghrelin can be metabolically active in an opposing manner to acyl-ghrelin to improve glycemic control. Furthermore, in vivo work has shown that desacyl-ghrelin alone does not alter food intake, but in keeping with the observed metabolic effects, attenuates acyl-ghrelin -induced food intake and arcuate nucleus neuronal activation [183,236,237]. It has also been suggested that desacyl-ghrelin acts independently of acyl-ghrelin via the hypothalamus to decrease food intake and gastric motility [9], and central desacyl-ghrelin administration was reported to increase food intake via activation of orexin neurons in the lateral hypothalamus [238]. It has been further demonstrated that intracerebroventricular and intravenous injections of desacyl-ghrelin disrupted fasted motor activity in the stomach [239]. For further information the reader is directed towards a comprehensive review by Soares and colleagues which summarise effects of both isoforms on the various systems and organs [240].

The pharmacokinetic parameters of infused acyl-ghrelin, desacyl-ghrelin, or a combination thereof in healthy subjects have been reported. The plasma half-life of acyl-ghrelin was 9–11 min after an intravenous infusion, whereas the half-life of total ghrelin (acyl-ghrelin + desacyl-ghrelin) was 35 min, indicating that desacyl-ghrelin has a slower clearance than acyl-ghrelin [217]. Similar estimates of half-lives have been reported elsewhere [209,212]. It is estimated that the ratio of des-acylated: acylated form of ghrelin in the plasma exceeds 9:1 [241,242,243]. However, during an infusion of acyl-ghrelin, the ratio of desacylated:acylated is 2:1. Interestingly, it was also shown that acyl-ghrelin infusion is responsible for an absolute increase in circulating plasma levels of desacyl-ghrelin [217]. This indicates that upon entry to the circulation, acyl-ghrelin is de-acylated, hence leading to an increase in desacyl-ghrelin which potentially counters the effects of acyl-ghrelin. Interestingly, in Prader-Willi syndrome, patients with an elevated ratio of acyl- to desacyl-ghrelin show pronounced hyperphagia and weight gain compared to those patients who display a normal acyl:desacyl ratio [244]. Therefore, acyl-ghrelin and desacyl-ghrelin not only exhibit different clearance rates from the circulation, but acyl-ghrelin is de-acylated in plasma. It is estimated that acyl-ghrelin accounts for only half of the increase in total ghrelin levels after dosing of acyl-ghrelin [209]. In this respect, active de-acylating enzymes have been identified in the circulation [245]. The ratio of desacyl-ghrelin:acyl-ghrelin can also change pending the metabolic state ie hunger can increase circulating acyl-ghrelin [229,246]. Given the proposed opposing effects of acyl- and desacyl-ghrelin, and the variable information in the literature vis-à-vis pharmacokinetic disposition, due consideration is warranted in the interpretation of trials to date.

### 3.5. Synthetic Ghrelin Ligands

It would appear therefore that the short half-life of acyl-ghrelin, the ubiquitous expression of GHSR-1a and the often overlooked presence of a functional antagonist in desacyl-ghrelin, leads to an unpredictable relationship between the pharmacokinetics and pharmacodynamics of ghrelin. Numerous synthetic ghrelin ligands have been developed over the years, all of which are more stable and exhibit a longer duration of action than native acyl-ghrelin [247,248]. From a pharmacokinetic perspective, increased half-life of synthetic compounds will lead to increased penetration into tissues and activation of the GHSR-1a for prolonged periods due to greater stability. In addition, synthetic derivatives are not converted to desacyl-ghrelin and avoid any potential counter effects. This therefore should lead to more predictable relationships of pharmacokinetics with pharmacodynamic effect.

Pharmacokinetic data is sparse for synthetic ligands, with many trials solely reporting on pharmacodynamic outcomes (Table 2). This is largely due to the focus of the field of research on ghrelin shifting over time. The first clinical studies mainly focus on ghrelin and ghrelin ligands as growth hormone (GH) secretagogues, thus solely measuring GH response and failing to measure serum ghrelin [249,250]. Indeed, it must be borne in mind that ghrelin had yet to be discovered for certain studies [251,252,253,254,255,256]. Originally, compounds such as Growth Hormone Releasing Peptide 6 (GHRP-6) and GHRP-2 were developed as somatotrophin secretagogues with the aim of treating GH deficiency syndromes such as pituitary dwarfism [251,252,255,256,257,258,259,260,261]. At the time of ghrelin’s discovery, focus shifted towards the possibility of exploiting these compounds for disorders of appetite [14,42,262,263]. With the increased appreciation of the role of ghrelin, research shifted to investigate its effects on the mesolimbic reward circuitry [29,264,265,266]. More recently, ghrelin agonists have been explored as gastrointestinal prokinetics to treat idiopathic and diabetic gastroparesis, as well as post-operative ileus [112,267,268]. Preclinical studies are thus difficult to directly compare due to variable approaches to dosing and vastly different experimental setups and outcome.

Nevertheless, the physiological mechanisms of appetite stimulation, body weight and other parameters for synthetic ligands (Table 2) are mediated through interaction with the GHSR-1a, and thus are broadly similar to ghrelin itself. Unfortunately, given the sparsity of comprehensive pharmacokinetic studies, many of parameters in Table 2 were taken from preclinical study data. No GHSR-1a antagonists or inverse agonists have been used clinically and there is a paucity of pharmacokinetic data available, hence they were not included in the scope for Table 2, however the reader is directed to a recent review for further information on these compounds [248]. Additionally, it is unwise to utilise pharmacodynamic outcomes as a surrogate measurement to compare ligand efficacy, due to heterogenous receptor-ligand interaction as discussed above [269]. For example, GH output is poorly correlated with orexigenic effect or body weight gain in vivo—stimulation of GH without affecting food intake has been demonstrated [258]. The agonist ulimorelin fails to elicit any GH release after both central and peripheral administration [270]. Anamorelin displays three times the potency of endogenous ghrelin in activating the ghrelin receptor in vitro [271]. However, it is noted this greater potency does not translate to greater in vivo levels of GH response [271]. Even minimal structural modifications of GH releasing peptide analogs affect the behavioural (food intake) but not GH-releasing properties of the analog [258]. Paradoxically, there have even been a number of reported GHSR-1a antagonists which display orexigenic effects. Although the antagonist BIM-28163 blocks ghrelin-induced GHSR-1a activation, and prevents GH secretion in vivo as a result, the compound elicits increases in food intake and body weight. However, this is thought to be potentially due to action at a receptor other than the GHSR-1a [272,273]. Furthermore, GSK1614343 also increased food intake and body weight in vivo, but knockout of the GHSR-1a abolished this effect, confirming that the antagonist was working via this receptor [274]. Antagonists with agonistic properties in vivo may be explained by biased agonism [275]. Vodnik and colleagues review several ligands which display biased agonism [248]. Individual drug-receptor interactions therefore determine distinct pharmacodynamic outcomes [276,277]. Different ligands can activate signalling cascades which may be more desirable and have the potential to be exploited for the development of more selective therapeutics [275]. This has led to examination of ligands, including inverse agonists, with selective effects for certain outputs. For example agonists for treating osteoporosis through GH secretion may have the adverse effect of increasing body weight [275]. Antagonists for GHSR-1a may be developed with the ability to decrease centrally-mediated food intake and adiposity, without inhibiting GH secretion. The potential of utilising biased agonism to achieve improved therapeutic efficacy warrants further investigation.

BBB penetration per se does not seem to be a key criterion for effecting changes to the centrally-mediated processes of appetite stimulation, growth hormone output or adipogenesis. This is probably due to a hijacking of the endogenous mechanisms of transport for ghrelin across the BBB and is in line with the literature on mechanism of CNS access of ghrelin discussed in the earlier parts of this review [188,190]. Despite its non-centrally penetrant action, anamorelin is in phase 3 trials for the treatment of cancer-anorexia-cachexia syndrome [323,324]. The compound elicits an orexigenic effect pointing to a central mechanism much in line with ghrelin’s homeostatic action, with a lack of traditional CNS penetration. This is also the case for other non-centrally penetrant compounds [258,259]. Given the expression of the GHSR-1a in less accessible brain areas, particularly in relation to incentive salience, there is an impetus to investigate BBB penetrability of ghrelin ligands further.

Preclinical work has already shown the potential benefits of BBB penetrant ghrelin agonists in other therapeutic areas. Activation of GHSR-1a in the spinal cord activates colonic motility. In the rat, severing the spinal cord at a thoracic level prevented defecation induced by the centrally penetrant agonist CP464709 [325]. Critically, this stimulation of colorectal activity was evident after *peripheral* administration of the ghrelin agonist, indicating a direct action on GHSR-1a in lumbosacral defecation centres. Furthermore, the lack of effect of peripheral ghrelin on the colon in vivo demonstrates the importance of BBB penetration [118]. GSK 894281 is an orally bioavailable BBB-penetrant ghrelin agonist which causes a prompt and dose-related output of faecal pellets after administration [326]. HM01 is another such agonist in preclinical trials as a colokinetic; again, its prokinetic action is attributed to its ability to cross the BBB and act on GHSR-1a’s present in the nerves of the lumbar section of the spinal cord [327,328,329,330].

Centrally penetrant GHSR-1a antagonists reduced body weight in diet-induced obese (DIO) mice when administered for 10 days, while also improving glucose tolerance [331,332]. Conversely, a non CNS-penetrating antagonist demonstrated comparatively mild effects on body weight, while retaining an effect on the peripherally regulated glucose tolerance. It has been postulated that the efficacy of these compounds on food intake and body weight appears to be correlated with their ability to antagonize central vs. peripheral GHSR-1a’s in different animal models [333]; YIL 870 and YIL 781 are quinazolinone-derived GHSR-1a antagonists which differ mainly in their ability to traverse the BBB. YIL 870 produces greater anorexigenic and weight reducing effects in diet-induced obese mice vs. the non-penetrant YIL 781, while both yielded a comparative improvement in glucose tolerance which has a peripheral element to its regulation [331]. Robust evidence thus shows that for antagonists to be effective in regulating body weight they need to cross the BBB. Pharmacological evaluation in obesity-induced rats revealed that a BBB penetrant inverse agonist for the GHSR-1a effectively reduced weight gain [334]. Ad libitum food intake was also reduced in mice treated with a BBB-penetrant inverse agonist (AZ-GHS-38) while a lack of efficacy was obtained in mice treated with a non- BBB-penetrant inverse agonist [335]. Therefore, a crucial determinant of the anti-obesogenic potential of GHSR-1a inverse agonists and antagonists is their ability to traverse the BBB.

The effect of ghrelin antagonists on the mesolimbic dopaminergic pathway has been investigated in the context of addictive-like behaviour. JMV 2959 is a centrally active GHSR-1a antagonist found to effectively reduce rewarding properties of addictive substances [336,337,338]. Systemic administration of JMV attenuated ghrelin-induced motivation to work for sugar pellet reward [338] in an operant conditioning paradigm. It was found that cocaine and amphetamine-induced place preference and extracellular accumbal dopamine were attenuated by administration of JMV 2959. This demonstrates a role for the GHSR-1a in the pathogenesis of addiction, while also suggesting the importance of ligand access to less accessible brain areas. These findings also generalise to opioid-induced dopamine release [336,339]. Notably, Jerlhag and colleagues have also concluded that BBB penetrant GHSR-1a antagonists may have potential in alcohol use disorders [340].

### 3.6. Hunger Is the Best Sauce—Targeting the Mesolimbic Reward Circuitry

The old adage that “hunger is the best sauce” may provide a potential novel approach for appetite modulation therapies—food becomes more appealing the hungrier we are [57]. This is an evolutionally-procured mechanism for survival in order to promote food intake beyond the immediate metabolic demand, to compensate for times of food scarcity [341]. The unravelling role of ghrelin and the expression of GHSR-1a in a number of brain areas associated with reward, meant that it became implicated in food-reward directed behaviour [264,265,338]. Consequently, the GHSR-1a may be a driver in the decision to eat palatable, calorie-dense foods, often beyond metabolic need. The role which ghrelin is purported to play at the interface between homeostatic and hedonic food intake regulation has been reviewed [26,28,29]. We have previously summarised recent experiments examining ghrelin’s effect on rewarding food intake and preference [26]. It is now generally accepted that food intake is the result of an integrated multi-process neuro-circuit, involving the cortex and critically, the mesolimbic dopaminergic system—therefore, targeting GHSR-1a in the midbrain reward system, with BBB-penetrant ligands, may hold novel therapeutic potential.

One of the key areas expressing the GHSR-1a in this respect is the ventral tegmental are (VTA). The importance of dopaminergic VTA outputs in feeding has been well established [342,343,344]. Central ghrelin administration recruits dopaminergic neurons in the VTA and results in an elevated dopaminergic tone in the nucleus accumbens (NAcc) of mice, while more targeted intra-VTA administration robustly increases the intake of both standard chow [17,345] and palatable food [265,346]. Incidentally, ghrelin administration into the medial prefrontal cortex also induces palatable-reward seeking behaviour in rats [347]. Microdialysis and electrophysiological studies in rodents have shown that peripheral ghrelin enhances dopaminergic neuronal firing, synapse formation and dopamine turnover in the NAcc. In animals, peripheral ghrelin treatment has increased locomotor activity and motivation to work for food, while also shifting food preference towards calorie dense and palatable foods [29,74,264,265,338,348]. Kawahara and colleagues showed that hunger in the absence of food creates an aversive neurocircuit in the reward pathway—dopamine outflow in the NAcc shell increased when food was present after injection, however decreased when no food was present [266]. Intraperitoneal administration of ghrelin decreases the firing of dopaminergic neurons in the VTA in food-deprived Wistar rats [349]. Therefore, peripheral ghrelin induced bimodal effects on the mesolimbic dopamine system depending on the food-consumptive status [266]. For further detailed discussion of the preclinical studies in this area the reader is guided towards recent reviews [57,62].

**Figure 2:**
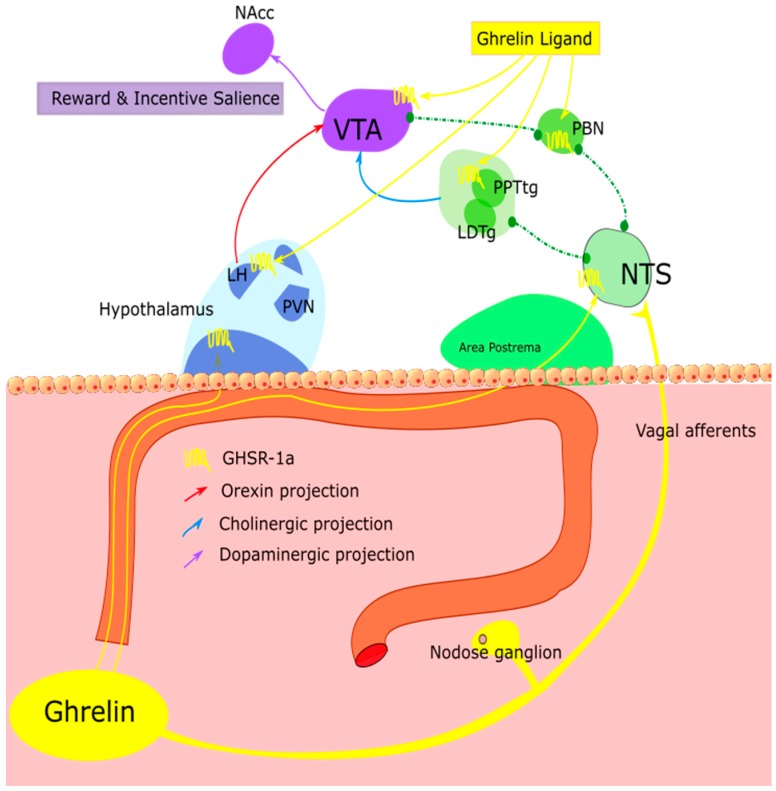
Direct and indirect access of ghrelin to the mesolimbic circuitry; the routes by which ghrelin and ghrelin ligands can traverse the blood-brain barrier (BBB). Direct activation of the mesolimbic circuitry can be attained by a centrally penetrant ghrelin agonist or by ghrelin which freely diffuses across the BBB. Indirect activation of mesolimbic circuitry is attained via the homeostatic mechanism through the “leaky” BBB capillaries at the median eminence and the area postrema. Ghrelin signalling initiating in the arcuate nucleus increases the rewarding value of food via orexin projections (red arrow) to the ventral tegmental area (VTA) from the lateral hypothalamus (LH). The nucleus tractus solitarius (NTS) displays connections with the hypothalamus, as well as the parabrachial nucleus (PBN), the laterodorsal tegmental area (LDTg) and pedunculopontine tegmental area (PPTtg), all of which have confirmed roles in either reward signalling (LDTg and PPTtg, blue arrow) or gustatory processes (PBN). Central penetration of ghrelin compounds may act directly on GHSR-1a expressed in these regions to modulate incentive salience of food (purple arrow).

There is thus ample evidence to suggest that peripheral ghrelin is able to exert an effect on less accessible brain regions associated with reward and motivation, such as the VTA (Figure 2). The mechanism by which peripheral ghrelin achieves access to other subcortical brain areas which are spatially separated from the circumventricular organs has been debated. It is now widely believed that ghrelin itself is not synthesized in the brain [105,106,188]. Jerlhag and colleagues have shown that ghrelin is able to access the VTA [350], while ghrelin has also been demonstrated to access the hippocampus [102]. Since these however, tracer studies using radio-labelled ghrelin have only been able to show that peripheral ghrelin reaches the arcuate nucleus at the level of the median eminence [351], and to a lesser extent the area postrema [106]. An evolutionally developed pathway has been argued to allow for selective transport of ghrelin across the BBB [190,352]. In vitro, human ghrelin exhibits saturable transport mechanics in the blood-to-brain as well as brain-to-blood directions in a rat cerebral microvessel endothelial model [353]. An in vivo mouse model reported findings consistent with this [190]. Indeed, many other endogenous substrates have inherited carrier mediated transport systems, such as glucose and insulin [354,355]. Furthermore, there is evidence to show that access of ghrelin to the brain via diffusion can increase or decrease depending on the physiological/metabolic backdrop or state of hunger [352]. Thus serum factors and physiological state are important determinants in the extent of the saturable ghrelin transport [352]. Therefore, it seems that central access of ghrelin may increase in calorie-deprived states.

The most likely mechanism of action of ghrelin in less accessible brain areas however, is through activation of neuronal populations via the permeable zones of the arcuate nucleus and the area postrema. From here, ghrelin acts to stimulate neuronal projections to other appetite centers not adjacent to the median eminence, such as the lateral hypothalamus [356,357]. The lateral hypothalamus (LH) is a key relay station for neuronal input to the VTA [358], and electrical stimulation of the LH induces voracious feeding even in well-fed animals [359]. It receives multiple excitatory and inhibitory inputs from both cortical and subcortical structures, however of particular note is input from the adjacent arcuate nucleus [360]. Differentially stimulating the neurons projecting from the arcuate nucleus to the LH proves that homeostatic energy demands are met by arcuate nucleus, but the LH is responsible for driving reward-motivated feeding [359]. VTA dopaminergic neurons are modulated by the selectively expressed orexin neuropeptides in the LH [361]. Thus, the LH and orexins play an important role in food and drug reward behaviours [362,363]. Importantly, elevated peripheral ghrelin levels are known to communicate with the VTA to increase the rewarding value of food in an orexin-dependent manner [74,364]. Therefore, in periods of hunger ghrelin is able to access the arcuate nucleus to stimulate homeostatic feeding, while the LH is concomitantly activated, aided by its close proximity and connections with the arcuate nucleus. The associated hedonic output is distinct from, yet intertwined with homeostatic feeding due to its arcuate nucleus-dependant stimulation.

Another brain area of note for appetite regulation is the parabrachial nucleus, which is located in the hindbrain near the NTS [365,366,367,368]. Like the arcuate nucleus, the NTS is spatially located near a permeable or “leaky” area of the BBB and sends glutamatergic signals to the parabrachial nucleus (PBN). Recent work has confirmed this region also receives GABAergic input from hypothalamic agouti-related peptide neurons [369]. The PBN is an important site for processing of gustatory sensory information, with lesions of this area leading to disruption of hedonic feeding and taste-reactivity patterns [367,370,371,372]. The PBN projects to several areas, notably the lateral hypothalamus and paraventricular hypothalamus, and ventral tegmental area [373,374,375,376]. Afferent signals to the paraventricular nucleus of the hypothalamus exist which may be involved in tuning the behavioural response to rewarding food [377]. Interestingly, the parabrachial nucleus itself expresses GHSR-1a and unsurprisingly this hedonic “hotspot” is therefore responsive to ghrelin treatment [378]. Consequently, it is postulated that in periods of hunger plasma ghrelin conveys NTS-dependent signalling to the PBN to exert an effect on feeding and reward behaviour [379,380].

Other areas such as the laterodorsal tegmental area and pedunculopontine tegmental neurons express GHSR-1a and elicit excitatory input to the VTA [350,381]. The pedunculopontine nucleus is implicated in the motivational effects of drugs and food [382]. Interestingly, in vitro work has demonstrated an excitatory effect of ghrelin on pedunculopontine neurons, suggesting a role in food reward [381,383]. The laterodorsal tegmental area increases dopamine output in the nucleus accumbens via the VTA, thereby confirming a GHSR-1a dependant role in reward [27,348].

### 3.7. Homeostatic “Gating” of the Reward System

Two decades of research on the effects of exogenous ghrelin has clearly demonstrated the function of GHSR-1a mediated signalling at the level of both homeostatic and non-homeostatic food intake. For homeostatic food intake it is clear that ghrelin has ready access to sites involved in feeding initiation through permeable brain capillaries and tanycytes [30], as well as vagal nerve communication [14,113,204,205]. Hedonic and motivational aspects of food intake have also been investigated mechanistically through site-specific administration [59,264,348]. The ability of ghrelin to communicate to less accessible GHSR-1a expressing brain areas such as the VTA, lateral hypothalamus and parabrachial nucleus suggests an indirect neural mechanism [194]. This is indicative of modulation or “gating” of the motivated response for food by systemic signals of energy homeostasis [384].

The midbrain reward system is thus heavily dependent on homeostatic appetite regulation in the arcuate nucleus and NTS, which constitute key “gatekeeping” structures to check the reward system under normal circumstances [385]. Perello and colleagues confirmed that neural connections between the arcuate nucleus and the VTA were responsible for peripheral ghrelin’s rewarding effect [74]. As we have seen however, preclinical and clinical studies have tended to use supra-physiological doses of ghrelin which may artificially increase delivery across the BBB by saturable transport processes [190] and diffusion from the circumventricular organs [188]. Elevated endogenous levels of ghrelin are able to elicit the same effects on hedonic aspects of food intake as high exogenous doses. This is due to the synergism of many systemic signals in energy-deprived states. The administration of high doses of a pleiotropic hormone may thus be leading to confounding compensatory mechanisms, particularly in relation to glucose homeostasis [148,364,386]. Directly targeting the GHSR-1a expressed in the reward circuitry through enhanced BBB penetration may hold therapeutic potential. One could hypothesise that a centrally-penetrant ghrelin agonist may affect mesolimbic dopamine levels and incentive valuation of food more directly than non-penetrating ghrelin agonists, or even ghrelin itself, through direct action on the GHSR-1a expressed on the lateral hypothalamus, parabrachial nuclei and the VTA. To the best of our knowledge this question has yet to be addressed experimentally.

## 4. Conclusions and Future Directions

Food intake and incentive valuation of food are centrally-mediated processes. Ghrelin or ghrelin ligands can access the brain from the periphery by circumventing the BBB at permeable locations adjacent to homeostatic appetite centres, and indirectly influence reward centres through neural connections stemming from these areas [74,384]. The importance of GHSR-1a signalling in the mesolimbic dopaminergic pathway as a barometer for the incentive salience of food has been well described. However, the action of GHSR-1a signalling on reward areas is closely intertwined with homeostasis, and is regulated in this respect [188,384]. The peripheral metabolic confounders in systemic ghrelin therapy, particularly relating to glucose homeostasis, may be contributing to the lack of successful preclinical moieties translating to clinical practice [387]. BBB-penetrant ghrelin agonists should bypass the homeostatic “gating” at the level of the arcuate nucleus and NTS. This means that they would act directly on GHSR-1a in less accessible brain areas associated with motivation and incentive valuation of food, such as the LH and VTA. Since the decision to eat is consciously made based on perceived palatability, centrally penetrating ghrelin agonists or indeed antagonists, could prove successful in manipulating top-down regulation of food intake.

## Figures and Tables

**Table 1:** Pharmacokinetic data available from clinical studies involving ghrelin.

Status	Dose of Infusion (Duration)	Fed Status	Form Assayed	Mean Serum Ghrelin (pmol/mL)	Average Fold Increase	Time Post-Dose (min)	Reference
Acylated	5 pmol/kg/min (180 min)	Overnight fasted	Total	1.32	Not reported	180 (*T*_max_)	[219]
Acylated	300 pmol/kg (Bolus) 1500 pmol/kg (Bolus)	Overnight fasted	Total and active	Total: 1.06 Acylated: 0.447 Total: 6.598 Acylated: 3.454	4.58 18.7 28.6 145.1	15 (*T*_max_) 15 (*T*_max_)	[209]
Acylated	3000 pmol/kg (Bolus)	Overnight fasted	Total	44.5	61	1	[220]
Acylated	5 pmol/kg/min (65 min) 15 pmol/kg/min (65 min) 25 pmol/kg/min (65 min)	Overnight fasted	Total and active	Total: 1.647 Acylated: 1.170 Total: 5.139 Acylated: 3.510 Total: 8.619 Acylated: 5.880	Not reported 118 Not reported 355 Not reported 594	45 (*T*_max_)	[217]
Acylated	84 pmol/kg (Bolus) + 5 pmol/kg/min (65 min)	Overnight fasted	Active and inactive	Acylated: 0.579 Desacylated: 0.350	44 17	30 (*T*_max_)	[217]
Desacylated	343 pmol/kg + 20.8 pmol/kg/min (65 min)	Overnight fasted	Active and inactive	Acylated: 0.006 Desacylated: 4.955	No change 233	Not specified	[217]
Acylated and Desacylated	Acylated: 84 pmol/kg (Bolus) + 5 pmol/kg/min (65 min) Desacylated: 343 pmol/kg + 20.8 pmol/kg/min (65 min)	Overnight fasted	Active and inactive	Acylated: 0.495 Desacylated: 4.644	54 272	Not specified	[217]
Acylated	1 pmol/kg/min (75 min) 5 pmol/kg/min (75 min)	Overnight fasted	Total	0.725 1.598	1.6 3.6	45 (*T*_max_) 45 (*T*_max_)	[58]
Acylated	1 pmol/kg/min (120 min) 5 pmol/kg/min (120 min)	Not specified	Total	0.958 4.087	3.54 15.13	90 90	[206]
Acylated	0.3 pmol/kg/min (300 min)	Fed	Active	0.057	2.4	210 (*T*_max_)	[216]
Acylated	7.5 pmol/kg/min (120 min) 15 pmol/kg/min (120 min)	Overnight fasted	Total	0.300 0.494	2 3	120 (*T*_max_) 120 (*T*_max_)	[221]
Acylated	3600 pmol/kg (Subcutaneous)	Overnight fasted	Total and active	Total: 0.988 Acylated: 0.355	5.15 10.23	15 (*T*_max_) 30 (*T*_max_)	[222]
Acylated	300 pmol/kg (Subcutaneous) 1500 pmol/kg 3000 pmol/kg	Overnight fasted	Total	~0.350 ~0.900 ~1.400	2 8 12	30 (*T*_max_)	[223]

**Table 2:** Ghrelin agonists used clinically. The half-life, oral bioavailability and centrally-mediated effects have been summarised. To date, no GHSR-1a antagonists have reached clinical trials.

Agonist	Class of Compound	Oral Bioavailability (Species)	Half Life	Centrally Regulated Parameters Reported
Growth Hormone Releasing Peptide 6 (GHRP-6)	Synthetic peptide	0.3% (Human) [247,278]	0.3 h [247]	Food intake [262], Body weight [262,279], Gastric emptying [119], Growth hormone [251,279]
Hexarelin	Synthetic peptide	<0.3% (Human) [280]	1.15 h [257,280]	Food intake [258], Growth velocity [281,282,283]
Pralmorelin (GHRP-2)	Synthetic peptide	Not reported, but has been dosed orally [284]	0.52 h [285]	Food intake [86,259], Growth hormone [284,285]
Alexamorelin	Synthetic peptide	Not reported	Not reported	Growth hormone [286]
Ipamorelin	Synthetic peptide	1%–6% (Rat, Dog) [287]	2 h [288]	Growth hormone [287,289], Body weight [287], Gastointestinal motility [290]
Capromorelin	Small molecule	65% [291] (Rat) [292]	2.4 h [291]	Growth hormone [291,293], Body weight [294], Gastric emptying [119]
Relamorelin	Synthetic peptide	Not reported	19.4 h [295]	Growth hormone [296], Food intake, Body weight [297,298,299], Gastric emptying [300,301]
Macimorelin	Small molecule	Not reported, but has been dosed orally [302,303]	3.8 h [304]	Growth hormone [302,305]
Tabimorelin	Synthetic peptide	30%–35% (Rat) [253,306]	20.8 h [307,308]	Growth hormone [306,307,308] Body weight [253]
Anamorelin	Small molecule	Not reported, but has been dosed orally [309,310,311]	7 h [309]	Growth hormone [309,312], Food intake [271,310,311,312]
Ibutamoren (MK-0677)	Small molecule	>60% (Dog) [254,313,314]	6 h [180]	Growth hormone [254,313,315], Body weight [316], Fat free mass [314]
Ulimorelin	Synthetic peptide	24% (Rat) [270]	1.6 h [317,318,319]	Growth hormone (no effect), Food intake, Gastrointestinal motility [270,317,319,320,321,322]

Enhancing efficacy through BBB penetration.

## Abbreviations

GHSR-1a	Growth Hormone Secretagogue Receptor-Type 1a
BBB	Blood Brain Barrier
GPCR	G-Protein Coupled Receptor
CNS	Central Nervous System
CSF	Cerebrospinal Fluid
NTS	Nucleus Tractus Solitarius
CCK	Cholecystokinin
GH	Growth Hormone
GHRP	Growth Hormone Releasing Peptide
DIO	Diet Induced Obesity
VTA	Ventral Tegmental Area
NAcc	Nucleus Accumbens
LH	Lateral Hypothalamus
PBN	Parabrachial Nucleus
